# Neuropsychiatric Symptoms Associated With Frontotemporal Atrophy in Older Adults Without Dementia

**DOI:** 10.1002/gps.70008

**Published:** 2024-12-01

**Authors:** Ioannis Liampas, Vasileios Siokas, Polyxeni Stamati, Panayiota Kyriakoulopoulou, Zisis Tsouris, Elli Zoupa, Vasiliki Folia, Constantine G. Lyketsos, Efthimios Dardiotis

**Affiliations:** ^1^ Department of Neurology University Hospital of Larissa School of Medicine University of Thessaly Larissa Greece; ^2^ School of Medicine University of Patras Patras Greece; ^3^ Larisa Day Care Center of People with Alzheimer's Disease Association for Regional Development and Mental Health (EPAPSY) Marousi Greece; ^4^ Lab of Cognitive Neuroscience School of Psychology Aristotle University of Thessaloniki Thessaloniki Greece; ^5^ Department of Psychiatry and Behavioral Sciences Johns Hopkins School of Medicine Baltimore Maryland USA

**Keywords:** aberrant motor behavior, apathy, appetite disorders, disinhibition, elation

## Abstract

**Objectives:**

We investigated the association between neuropsychiatric symptoms (NPS) and frontotemporal atrophy (FTA) in older adults without dementia. We hypothesized that the odds of having NPS would be increased in the presence of FTA.

**Methods:**

NACC participants ≥ 50 years old with available data on FTA were considered for eligibility. Those with a diagnosis of mild cognitive impairment (MCI) and those who were cognitively unimpaired (CU) were separately analyzed. NPS were quantified on the Neuropsychiatric Inventory Questionnaire. Binary logistic regression models estimated the association (odds ratios and 95% confidence intervals are provided) between FTA and having each of 11 NPS (psychotic symptoms were grouped together) in CU and MCI individuals.

**Results:**

FTA data were available for 3165 participants with MCI and 4051 CU: 207 and 55 had FTA on structural MRI studies, respectively. In the MCI group, the presence of FTA was associated with higher odds of having elation [2.42(1.33–4.40), *p* = 0.004], aberrant motor behavior [2.43(1.61–3.69), *p* < 0.001], appetite disorders [2.15(1.52–3.04), *p* < 0.001], apathy [2.05(1.48–2.85), *p* < 0.001] and disinhibition [2.02(1.38–2.96), *p* < 0.001]. The odds of having specific NPS were not significantly elevated in CU individuals with FTA. Of note, the size and direction of the associations were indicative of a potential relationship between FTA and specific NPS (most notably elation, aberrant motor behavior, appetite disorders and anxiety); in light of the small number of CU individuals with FTA we believe this analysis was underpowered and obscured several true associations.

**Conclusions:**

FTA was associated with higher odds of some NPS in older adults with MCI but not with normal cognition.

**Summary**

- The average number and severity of NPS was greater in FTA+ compared to FTA− participants with MCI.
- The average number and severity of NPS was similar in FTA+ and FTA− participants with normal cognition.
- The odds of having aberrant motor behaviors, elation, appetite disorders, apathy and disinhibition were significantly higher in the FTA+ MCI group.
- The odds of having specific NPS were not significantly higher in the FTA+ CU group.

## 1 Introduction

Frontotemporal dementia (FTD) is a major neurocognitive disorder with two common phenotypic presentations [1]: primary progressive aphasia (PPA) with early prominent language impairment [1, 2] and the behavioral variant (bvFTD) with early alterations in emotion, personality and executive function [1, 2]. Frontal and/or anterior temporal atrophy on magnetic resonance imaging (MRI) studies or hypometabolism on fluro‐deoxy‐glucose positron emission tomography (FDG‐PET) are imaging markers of bvFTD [3, 4]. Apart from these presentations, additional entities included in the spectrum of frontotemporal lobar degeneration (FTLD) are FTD− motor neuron disease (MND), corticobasal degeneration (CBD) and progressive supranuclear palsy (PSP) [1].

Neuropsychiatric symptoms (NPS) are very common in older adults with mild cognitive impairment (MCI) and almost universal in those with dementia [5, 6]. In cognitively unimpaired (CU) individuals, NPS are associated with worse cognitive performance [7], steeper cognitive trajectories [8] and increased risk of Alzheimer's disease (AD) or non‐AD dementia [9]. Similarly, in older adults with MCI, NPS are linked to more precipitous cognitive changes [10] and elevated risk of future dementia [11], while among those with dementia, NPS are a forerunner of more abrupt cognitive decline [12] among other unfavorable outcomes [13]. Therefore, the presence of NPS in older adults should be regarded as a harbinger of cognitive decline throughout the normal aging‐dementia continuum. Although the majority of published evidence on NPS relates to Alzheimer disease, there is growing evidence of associations between NPS and disease progression in FTD [9]. In the continuum of healthy aging—FTD, Taragano and colleagues introduced the construct of mild behavioral impairment (MBI)—the neuropsychiatric equivalent of MCI, as a transitional stage between normal aging and dementia which confers greater risk of incident dementia than MCI [14].

The predominant hypothesis suggests that the association between NPS and cognitive decline probably reflects the relationship of NPS with undergoing neuropathological alterations [15]. Different NPS have been related to different neurodegenerative processes and by extension to heterogeneous cognitive trajectories and progression to different neurocognitive entities; for instance, psychosis has been linked to neuritic plaques, neurofibrillary tangles and Lewy body disease (and in turn to AD and Lewy body dementia ‐LBD), whereas agitation and aggression have been associated with TDP‐43 pathology (a common substrate of FTD) [16]. While many studies have examined associations between cortical atrophy and NPS among older adults with dementia, only a few have investigated this relationship in individuals without dementia (MCI or normal cognition). Even fewer studies have focused on frontal and/or anterior temporal atrophy, in particular [17, 18]. Prior studies featured small samples and often did not account for the confounding of neurocognitive status. The aim of this analysis was to examine in older adults without dementia whether frontotemporal atrophy (FTA) affects the odds of different NPS. Based on published literature, we hypothesized that older adults with evidence of frontal and/or anterior temporal atrophy in structural MRI studies would have higher odds of having individual NPS.

## 2 Methods and Materials

This cross‐sectional analysis capitalized on data from the ongoing Uniform Data Set (UDS). UDS is a central repository of multidisciplinary, longitudinally collected data in National Institute on Aging–funded Alzheimer's Disease Research Centers (ADRCs) across the United States [19, 20, 21, 22]. UDS was instituted in 2005 and has since been stewarded by the National Alzheimer's Coordinating Center (NACC). Clinician‐, self‐ and family‐referred volunteers or actively recruited individuals with a cognitive status ranging from unimpaired cognition to dementia are enrolled according to each ADRC's discrete protocol. Standardized evaluations take place approximately every year. Participants or surrogates provide informed consent before participation. All procedures are overseen by Institutional Review Boards at each ADRC and performed in accordance with the ethical standards laid down in the declaration of Helsinki and its later amendments. For further information on the NACC database, please visit https://naccdata.org/.

### 2.1 Eligibility Criteria and Diagnostic Procedures

This analysis included UDS data from the December 2022 data freeze, collected from a total of 46 ADRCs. Participants ≥ 50 years old with data on FTA status derived from structural MRI studies (only the 1st visit with available data was considered for eligibility—neuropsychiatric assessments from the same visit were capitalized on) and a concurrent diagnosis of MCI or CU, were included. Cognitive diagnoses were established by either expert consensus panels (in the majority of cases) or single physicians (i.e., those who conducted the examination), according to each ADRC's discrete protocol. MCI and dementia were diagnosed using standard clinical criteria [23, 24, 25, 26, 27, 28]. Normal cognition was defined by the absence of a diagnosis of dementia, MCI or cognitive impairment not MCI (i.e., participants with cognitive impairment who did not clearly fit into the dementia or MCI categories).

### 2.2 Measurement of NPS (Outcome Variable)

The Neuropsychiatric Inventory Questionnaire (NPI‐Q) is an informant administered, widely used tool for the evaluation of NPS in dementia research [29]. NPI‐Q evaluates 12 domains: delusions, hallucinations, agitation/aggression, depression/dysphoria, anxiety, elation/euphoria, apathy/indifference, disinhibition, irritability/lability, aberrant motor behavior, night‐time behaviors, and eating behaviors. Informants initially report the presence or absence of cardinal symptomatology for each domain in the month preceding the examination and subsequently rate the severity of any symptoms according to a 3‐point severity scale: mild (noticeable, but not a significant change); moderate (significant, but not a dramatic change); or severe (very marked or prominent; a dramatic change) [30, 31]. For the current analysis, participants were dichotomized by presence of each NPS (0: absent; 1: present). Delusions and hallucinations were grouped together (psychotic symptoms) owing to their very low prevalence. Two additional composite NPS indices were analyzed: total number of NPS (0–11) and total NPS severity (0–22). For the latter, absence of NPS was scored with 0, mild symptomatology conferred 1 point and moderate to severe symptomatology conferred 2 points.

### 2.3 Fronto‐Temporal Atrophy (FTA) (Predictor Variable)

Based on presence or absence of FTA, two groups of participants were defined with (FTA+) and without frontal and/or anterior temporal atrophy (FTA−). UDS's MRIs are best described as a convenience sample submitted by certain ADCs. Imaging data collection and acquisition protocols varied by ADC. Additionally, the lack of “central”, standardized evaluation introduced further heterogeneity in assessments. In light of the lack of central—uniform and blinded assessments, variability should be expected among different assessors in the confirmation of FTA.

### 2.4 Statistical Analysis

Demographics of the two cognitive groups (CU and MCI) were compared (FTA+ vs. FTA−) using independent sample *t*‐tests (scale variables) and Pearson chi‐squared tests (categorical variables). Similarly, the frequencies of different NPS were compared between the two groups. Next, binary logistic regression models estimated the odds of having each NPS in people with CU or MCI comparing FTA+ to FTA−. Models were adjusted for age, years of education, sex, race and MCI status. In particular, the five MCI subtypes ‐treated as dichotomous variables‐ were inserted in every model, that is, MCI memory: yes/no; MCI language: yes/no; MCI executive function: yes/no; MCI visuospatial: yes/no; MCI attention: yes/no. Odds ratios and 95% confidence intervals are provided.

The second analysis ‐involving CU participants‐ followed to the same analytical approach. Models were again adjusted for age, years of education, sex and race. In this case, to account for the potential confounding of cognition, models were additionally adjusted for mini‐mental state examination (MMSE) scores. The Montreal Cognitive Assessment (MoCA) was utilized instead of MMSE in the last (3rd) version of UDS. To limit the amount of missing data, MoCA values were converted to MMSE scores according to the detailed conversion tables provided by a NACC crosswalk study [32, 33].

Finally, composite NPS measures (scale variables: total number of NPS—total NPS severity) were sequentially inserted into univariate general linear models (GLMs) as dependent variables. GLMs were adjusted for the same covariates as before. Separate analyses were performed for the CU and MCI groups.

Statistical analyses were performed using the IBM SPSS Statistics Software Version 26 (Chicago, IL, USA). Due to multiple comparisons (11 per participant set), the stricter threshold of *α* = 0.004 was implemented for the revelation of statistical significance.

## 3 Results

### 3.1 FTA and NPS in MCI Participants

Three thousand one hundred sixty five participants with MCI and data on FTA were eligible for analysis. Of these, 207 had FTA and 2958 did not. Differences between those with and without FTA are in Table 1. The sample predominantly comprised older, well‐educated, Caucasians. Individuals with MCI and FTA were younger, more often Caucasian and had predominant executive function, language, or attention deficits. Participants with MCI but no FTA were more often African‐American. Amnestic MCI was more prevalent in those without FTA. Participants with MCI and FTA had a higher prevalence of most NPS. Elation was > 3× more common than in the FTA− group, aberrant motor behaviors were ∼3×, disinhibition, appetite disorders and apathy ∼2×, while depression, irritability and night‐time behaviors were also slightly more prevalent in FTA+ (Table 2). Differences in frequency of anxiety, agitation and psychosis were not significant levels in the two MCI groups.

**TABLE 1:** Baseline comparison of older individuals with mild cognitive impairment by frontotemporal atrophy (FTA) status.

Variable	Without FTA (*n* = 2958)	With FTA (*n* = 207)	*p*‐value
Age in years	73.16 ± 8.74	69.65 ± 8.66	**<** **0.001**
Formal education in years	16.02 ± 3.11	16.09 ± 3.28	0.482
Sex (male/female)	1486/1472 (50.2/49.8%)	107/100 (51.7/48.3%)	0.686
Race (Caucasian/African American/other)	2486/311/134 (84.8/10.6/4.6%)	186/3/14 (91.6/1.5/6.9%)	**<** **0.001**
MCI—memory (No/Yes)	593/2365 (20.0/80.0%)	98/109 (47.3/52.7%)	**<** **0.001**
MCI—language (No/Yes)	2053/905 (69.4/30.6%)	98/109 (47.3/52.7%)	**<** **0.001**
MCI—executive function (No/Yes)	1566/1392 (52.9/47.1%)	71/136 (34.3/65.7%)	**<** **0.001**
MCI—attention (No/Yes)	2397/561 (81.0/19.0%)	151/56 (72.9/27.1%)	**0.005**
MCI—visuospatial skills (No/Yes)	2518/440 (85.1/14.9%)	180/27 (87.0/13.0%)	0.473

*Note:* Bold denotes statistically significant differences between the two groups.

Abbreviation: MCI, mild cognitive impairment.

**TABLE 2:** Frequencies of neuropsychiatric symptoms by frontotemporal atrophy (FTA) status among older adults with mild cognitive impairment.

Variable	Without FTA (*n* = 2958)	With FTA (*n* = 207)	*p*‐value
Psychotic symptoms (No/Yes)	2682/147 (94.8/5.2%)	183/8 (95.8/4.2%)	0.541
Depression (No/Yes)	1902/920 (67.4/32.6%)	104/87 (54.5/45.5%)	**<** **0.001**
Anxiety (No/Yes)	1914/910 (67.8/32.2%)	120/71 (62.8/37.2%)	0.158
Agitation (No/Yes)	2351/478 (83.1/16.9%)	149/42 (78.0/22.0%)	0.071
Disinhibition (No/Yes)	2523/303 (89.3/10.7%)	147/44 (77.0/23.0%)	**<** **0.001**
Irritability (No/Yes)	1928/902 (68.1/31.9%)	112/79 (58.6/41.4%)	**0.007**
Elation (No/Yes)	2754/75 (97.3/2.7%)	174/17 (91.1/8.9%)	**<** **0.001**
Motor symptoms (No/Yes)	2627/199 (93.0/7.0%)	154/37 (80.6/19.4%)	**<** **0.001**
Apathy (No/Yes)	2260/570 (79.9/20.1%)	116/74 (61.1/38.9%)	**<** **0.001**
Appetite disorders (No/Yes)	2385/440 (84.4/15.6%)	132/58 (69.5/30.5%)	**<** **0.001**
Night‐time behaviors (No/Yes)	2048/721 (74.0/26.0%)	122/63 (65.9/34.1%)	**0.017**

*Note:* Bold denotes statistically significant between differences between the two groups.

After adjusting for age, education, sex, race and MCI subtypes, the average number of NPS was 2.12 (1.94–2.29) in FTA− and 2.87 (2.54–3.21) in FTA+ MCI participants [Mean difference (MD) = 0.76 (0.44–1.08), *p* < 0.001]. Average NPS severity was also greater in those with [4.15 (3.63–4.68)] versus those without [2.83 (2.56–3.11)] FTA [MD = 1.32 (0.83–1.82), *p* < 0.001].

After adjusting for age, education, sex, race and MCI status, the odds of having aberrant motor behaviors (*p* < 0.001) or elation (*p* = 0.004) were ∼2.4x higher in the FTA+ group (Table 3). The odds of appetite disorders (*p* < 0.001), apathy (*p* < 0.001) or disinhibition (*p* < 0.001) were > 2.0x higher. The remaining NPS were not related to FTA.

**TABLE 3:** Odds of having neuropsychiatric symptoms by frontotemporal atrophy status among older adults with mild cognitive impairment (MCI). The group without atrophy was used as reference.

Variable	Odds ratio	95% confidence interval	*p*‐value
Adjusted analyses			
Psychotic symptoms	0.60	0.28–1.28	0.184
Depression	1.40	1.02–1.92	0.036
Anxiety	1.03	0.75–1.56	0.855
Agitation	1.23	0.85–1.80	0.274
Disinhibition	**2.02**	**1.38–2.96**	**<** **0.001**
Irritability	1.33	0.97–1.82	0.080
Elation	**2.42**	**1.33–4.40**	**0.004**
Motor symptoms	**2.43**	**1.61–3.69**	**<** **0.001**
Apathy	**2.05**	**1.48–2.85**	**<** **0.001**
Appetite disorders	**2.15**	**1.52–3.04**	**<** **0.001**
Night‐time behaviors	1.09	0.88–1.40	0.629
Unadjusted analyses			
Psychotic symptoms	0.80	0.39–1.65	0.542
Depression	**1.73**	**1.29–2.32**	**<** **0.001**
Anxiety	1.24	0.92–1.69	0.158
Agitation	1.39	0.97–1.98	0.072
Disinhibition	**2.49**	**1.74–3.56**	**<** **0.001**
Irritability	1.51	1.12–2.03	0.007
Elation	**3.59**	**2.07–6.21**	**<** **0.001**
Motor symptoms	**3.17**	**2.16–4.69**	**<** **0.001**
Apathy	**2.53**	**1.86–3.43**	**<** **0.001**
Appetite disorders	**2.38**	**1.72–3.30**	**<** **0.001**
Night‐time behaviors	1.47	1.07–2.01	0.017

*Note:* Bold denotes statistically significant between differences between the two groups; analyses were adjusted for age, education, MCI subtypes, sex and race.

### 3.2 FTA and NPS in CU Participants

Four thousand and fifty one CU participants had data on FTA. Of these, 55 had FTA and 3996 did not. No demographic or cognitive differences were evident between those with and without FTA (Table 4). FTA+ CU participants had a higher prevalence of elation (4.1% vs. 0.8%) and anxiety (24.5% vs. 14.1%) compared to those who were FTA−. Most NPS were more prevalent in the former group (apart from psychotic symptoms), but differences were not significant probably due to the very small size of the group with FTA (Table 5).

**TABLE 4:** Baseline comparison of older, cognitively unimpaired individuals by frontotemporal atrophy (FTA) status.

Variable	Without FTA (*n* = 3996)	With FTA (*n* = 55)	*p*‐value
Age in years	69.73 ± 8.68	69.02 ± 9.60	0.547
Formal education in years	16.29 ± 2.80	16.67 ± 2.99	0.318
Mini‐mental state‐examination score	29.32 ± 1.24	29.12 ± 1.52	0.244
Sex (male/female)	1390/2606 (34.8/65.2%)	22/33 (40.0/60.0%)	0.420
Race (Caucasian/African American/other)	3255/496/228 (81.8/12.5/5.7%)	44/7/4 (80.0/12.7/7.3%)	0.883

**TABLE 5:** Neuropsychiatric symptoms by frontotemporal atrophy (FTA) status among older, cognitively unimpaired adults.

Variable	Without FTA (*n* = 3996)	With FTA (*n* = 55)	*p*‐value
Psychotic symptoms (No/Yes)	3707/25 (99.3/0.7%)	49/0 (100.0/0.0%)	0.565
Depression (No/Yes)	3153/570 (84.7/15.3%)	37/12 (75.5/24.5%)	0.077
Anxiety (No/Yes)	3198/523 (85.9/14.1%)	37/12 (75.5/24.5%)	**0.038**
Agitation (No/Yes)	3536/193 (94.8/5.2%)	46/3 (93.9/6.1%)	0.767
Disinhibition (No/Yes)	3622/103 (97.2/2.8%)	47/2 (95.9/4.1%)	0.578
Irritability (No/Yes)	3256/471 (87.4/12.6%)	40/9 (81.6/18.4%)	0.232
Elation (No/Yes)	3698/47 (99.2/0.8%)	47/2 (95.9/4.1%)	**0.015**
Motor symptoms (No/Yes)	3659/62 (98.3/1.7%)	47/2 (95.9/4.1%)	0.193
Apathy (No/Yes)	3520/202 (94.6/5.4%)	45/4 (91.8/8.2%)	0.402
Appetite disorders (No/Yes)	3525/190 (94.9/5.1%)	44/5 (89.8/10.2%)	0.110
Night‐time behaviors (No/Yes)	3165/468 (87.1/12.9%)	37/10 (78.7/21.3%)	0.089

*Note:* Bold denotes statistically significant differences between the two groups.

After adjusting for age, education, sex, race and MMSE scores, the average number of NPS was 1.29 (0.88–1.70) in those with and 0.86 (0.77–0.95) in those without FTA. The total number of NPS was comparable in CU individuals with and without FTA [MD = 0.43 (0.02–0.84), *p* = 0.040]. Average NPS severity was also similar in those with FTA [1.66 (1.06–2.27)] versus those without FTA [1.15 (1.02–1.28)] [0.51 (−0.09–1.11), *p* = 0.095].

After adjusting for age, education, sex, race and MMSE scores, the odds of having specific NPS [most notably elation (∼4.8× higher), aberrant motor behaviors (∼2.5× higher), appetite disorders (∼2.2× higher) and anxiety (∼2.2× higher)] were higher but not significantly so in the FTA+ CU group (Table 6). Given the consistency in the nature and size of associations, in light of the small number of FTA+ CU individuals we believe this analysis was underpowered and obscured several true associations.

**TABLE 6:** Odds of having neuropsychiatric symptoms by frontotemporal atrophy (FTA) status among older, cognitively unimpaired adults: the group without atrophy was used as reference.

Variable	Odds ratio	95% confidence interval	*p*‐value
Adjusted analyses			
Psychotic symptoms	NA	NA	0.998
Depression	1.87	0.95–3.67	0.070
Anxiety	2.15	1.10–4.19	0.025
Agitation	1.16	0.35–3.81	0.805
Disinhibition	1.34	0.32–5.63	0.694
Irritability	1.54	0.73–3.26	0.258
Elation	4.81	1.10–21.11	0.038
Motor symptoms	2.54	0.59–10.94	0.210
Apathy	1.51	0.53–4.31	0.437
Appetite disorders	2.20	0.85–5.71	0.105
Night‐time behaviors	1.66	0.78–3.48	0.182
Unadjusted analyses			
Psychotic symptoms	NA	NA	0.998
Depression	1.79	0.93–3.46	0.081
Anxiety	1.98	1.03–3.83	0.041
Agitation	1.20	0.37–3.88	0.767
Disinhibition	1.50	0.36–6.24	0.580
Irritability	1.56	0.75–3.23	0.442
Elation	5.08	1.18–21.83	0.029
Motor symptoms	2.51	0.60–10.57	0.209
Apathy	1.55	0.55–4.35	0.406
Appetite disorders	2.11	0.83–5.38	0.119
Night‐time behaviors	1.83	0.90–3.70	0.094

*Note:* Bold denotes statistically significant differences between the two groups; analyses were adjusted for age, education, MMSE scores, sex and race; NA: non‐applicable—no participant with FTA had psychotic symptoms.

## 4 Discussion

We report that older adults with MCI and evidence of frontal and/or anterior temporal atrophy in structural MRI studies had higher odds of having NPS compared to individuals without FTA, in particular, elation, aberrant motor behaviors, apathy, appetite disorders and disinhibition. Although our estimations were underpowered, older CU adults with FTA also appeared to have greater odds of NPS especially elation, motor symptoms, appetite disorders and anxiety‐ compared to FTA− CU controls. These estimations accounted for important demographic factors, as well as cognitive functioning. These findings align with the hypothesis that underlying FTA contributes to the overall neuropsychiatric burden, independent of cognitive impairment.

The core neuropsychiatric manifestations of bvFTD involve disinhibition, loss of sympathy/empathy, apathy/inertia, hyperorality and perseverative/compulsive behaviors (reflected on the motor symptoms item of the NPI‐Q) [3]. Considering NPS (among other clinical manifestations) may precede dementia onset, these associations may extend to the prodromal stages of the disorder. Apart from FTD, these symptoms (especially apathy and disinhibition) may elevate the risk of other major neurocognitive entities such as AD and LBD [9]. Nevertheless, the estimated effect sizes tend to be smaller and the pre‐diagnostic constellation of neurocognitive and motor symptoms characterizing these disorders may facilitate their discrimination from prodromal FTD [34, 35, 36].

Limited previous research has addressed the association between FTA and NPS using a more quantitative approach [37]. Reduced volumes in the gyrus rectus, medial frontal cortex, subcallosal area, superior and inferior frontal gyrus have been related to disinhibition in a sample of older adults with MCI, AD and bvFTD. Medial orbital and inferior frontal gyri atrophy have been linked to elation. Frontal pole and subcallosal area atrophy have been associated with aberrant motor behaviors. Asymmetrical atrophy patterns have been also related to distinct NPS in bvFTD [38]. Right‐lateralization has been associated with appetite—eating disorders and psychotic manifestations, whereas ventral‐dorsal predominance of atrophy correlated with anxiety, elation and disinhibition; agitation, irritability, and depression were linked to lack of asymmetry in FTA. Again, quantitative studies on FTA have not particularly focused on non‐demented individuals; therefore, additional research is warranted in this group.

The current study offers additional indirect evidence that FTA (and by extension underlying FTD pathology) overwhelms the associations of other common neurodegenerative pathologies with NPS. Neuropathologic alterations are almost universal in older adults with MCI. AD pathology predominates with vascular and Lewy body pathologies not infrequent [39, 40]. Among the well‐established relationships of these neuropathologies with NPS, amyloid pathology is associated with affective symptoms, apathy, night‐time behaviors and delusions. Further LBD pathology has been linked to hallucinations, affective symptoms and apathy, while white matter hyperintensities relate to symptoms such as elation, disinhibition and agitation [36, 41, 42, 43, 44, 45]. According to our findings, no NPS were less prevalent in individuals with MCI and FTA (only psychosis was relatively less common, but results were insignificant), whereas elation, motor symptoms, apathy, appetite disorders and disinhibition were more than twice as common. These findings suggest that FTD pathology may drive the manifestation of NPS significantly more than other common neurodegenerative alterations.

On the other hand, we failed to reveal a relationship between FTA and NPS in CU older individuals. Although our results were insignificant, considering the consistency in the direction and size of estimated effects, we believe this analysis was underpowered (very small number of FTA + CU individuals). However, we have to point out that alternative explanations may apply. First, the degree of brain atrophy is probably inferior in CU individuals and may be insufficient to set off any neuropsychiatric manifestations. Secondly, the unaccounted presence of co‐existing neuropathologies may drive our estimations, especially in the MCI group. Therefore, future research focusing on non‐demented individuals ought to address the existence of co‐pathologies as well as account for quantitative considerations.

The early identification of individuals with emerging neurodegenerative conditions is gaining more and more interest. Ongoing research (and clinical practice) emphasizes the early identification of high‐risk people for the implementation of preventive interventions during the prodromal stage of dementia [46]. Along with NPS, other clinical—most notably neuropsychological or motor—manifestations have been associated with specific neuropathological changes [47, 48]. Considering the well‐established relationship between cognitive performance and motor signs with incident dementia, their combination with neuropsychiatric assessments could potentially enhance the overall prognostic properties of prodromal symptomatology [49, 50, 51]. The use of clinical manifestations as an initial filter in the selection of candidates for more sophisticated biomarker investigations may limit the unwise implementation of interventional and/or costly procedures such as lumbar puncture and positron emission tomography scan. In other words, understanding associations between underlying neuropathology and prodromal clinical manifestations will facilitate the accurate choice of more sophisticated examinations and interventions.

This study has several strengths including the large sample of individuals with available MRI studies and the adequate number of those with FTA in the MCI group. The NPI‐Q was uniformly used to assess the presence of NPS. The neurocognitive status of participants (along with important demographic confounders) was accounted for in the analytical part of the article. The analysis has several weaknesses, as well. First, the number of certain NPS, as well as the number of older adults with FTA in the CU group, was small, such that some comparisons were underpowered. This is reflected in the large precision estimates of our findings and may have obscured several non‐trivial associations. Second, although several crucial factors and covariates were taken into account, the findings may have been driven by residual confounding or the non‐trivial proportion of missing data [52, 53]. Third, the presence or absence of FTA was not uniformly assessed by a central, blinded evaluator (or group of evaluators). Variability should be expected among different assessors in the confirmation of FTA. In addition, we did not correct our findings for multiple comparisons to retain statistical power despite the low frequency of certain NPS, and the low number of FTA+ people especially in the CU group. Nevertheless, in view of the strong associations in the MCI group (*p* < 0.001 for appetite disorders, apathy, motor symptoms and disinhibition), we are confident that at least some results reflect true associations. Moreover, we did not include additional imaging biomarkers, such as global or parietal atrophy, hippocampal volumes, and so on. Finally, another limitation is the observational nature of our study. Hence, it is not possible to make etiologic inferences about NPS and frontotemporal atrophy.

### 4.1 Conclusions

Frontal and/or anterior temporal atrophy in structural MRI studies is related to higher odds of NPS among older adults with MCI, especially aberrant motor behaviors, elation, appetite disorders, disinhibition, apathy and depression. These associations may extend to cognitively unimpaired people; however, additional studies involving larger samples are warranted to confirm this relationship. Future studies using longitudinal and quantitative approaches (to examine the association between frontotemporal volumes and incident NPS) may provide additional insight into the causative nature of this relationship.

## Author Contributions

I.L.: original draft preparation, data curation, formal analysis, design of the study, interpretation of data, and review & editing of manuscript; V.S., P.S., Z.T., A.P. & E.Z.: data curation, validation, review & editing of manuscript; V.F., C.G.L. & E.D.: conceptualization, formulation of research question, design of the study, supervision, review & editing.

## Ethics Statement

This cross‐sectional analysis capitalized on data from the ongoing Uniform Data Set (UDS). UDS is a central repository of longitudinally collected data in National Institute on Aging–funded Alzheimer's Disease Research Centers (ADRCs) across the United States. UDS is stewarded by the National Alzheimer's Coordinating Center (NACC). Participants are enrolled according to each ADRC's discrete protocol. All procedures are overseen by Institutional Review Boards at each ADRC and performed in accordance with the ethical standards laid down in the declaration of Helsinki and its later amendments. For further information on the NACC database, please visit https://naccdata.org/.

## Conflicts of Interest

The authors declare no conflicts of interest.

## Data Availability

For further information on access to the NACC database, please contact NACC (contact details can be found at https://naccdata.org/).
